# Logistic Regression Model for Determination of the Age of Brown Hare (*Lepus europaeus* Pall.) Based on Body Weight

**DOI:** 10.3390/ani12040529

**Published:** 2022-02-21

**Authors:** Marian Flis, Piotr Czyżowski, Sławomir Beeger, Bogusław Rataj, Mirosław Karpiński

**Affiliations:** 1 Department of Animal Ethology and Wildlife Management, University of Life Sciences in Lublin, Akademicka 13, 20-950 Lublin, Poland; marian.flis@up.lublin.pl (M.F.); piotr.czyzowski@up.lublin.pl (P.C.); slawomir.beeger@up.lublin.pl (S.B.); 2 Polish Hunting Association, District Board Nowy Sącz, 33-300 Nowy Sącz, Poland; brataj66@gmail.com

**Keywords:** brown hare, *Lepus europaeus*, body weight, lens weight, age determination

## Abstract

**Simple Summary:**

Because the determination of the age of brown hares (*Lepus europaeus*) is difficult, we developed a method for age determination on the basis of animal body weight. On the basis of the results of postmortem analysis of body weight and eye-lens weight, which provide reliable information on the change in these parameters with age, a logistic regression model was developed to classify brown hares as either juveniles (up to one year old) or adults (more than one year old), with a body weight of 4.227 kg as the limiting interval. With an accuracy of 95%, this noninvasive method can be used in both scientific research and population management.

**Abstract:**

We developed an algorithm to classify brown hares into two age classes, juveniles (up to 1 year old) and adults (over 1 year old), based on body weight, which can be determined by both the examination of live animals and postmortem analysis. Considering the strong correlation between lens weight and carcass weight, we assumed that hares could be classified into one of the two age groups based only on carcass weight, using a logistic regression model. Using logistic regression, a model was constructed to assess the age of hares based on their body weight. For comparison with the current age-assessment method based on the dry lens weight, a logistic regression classifying the hares based on the dry lens weight was performed as well. The results of the study facilitated the development of a method to classify hares into age groups based on body weight. The proposed approach is innovative, as it allows for the determination of the age of not only culled (postmortem) but also live hares. The method is easy and does not require laboratory tests; hence, the results can be used immediately following evaluation. This method allows hares to be categorized into two age groups (juveniles and adults). With an accuracy of 97.52% and 95.45% in the case of juvenile and adult hares, respectively, the proposed approach can be widely used both in population management and scientific research.

## 1. Introduction

In the management of hunting practices and the management of wild animal populations, animal welfare must be constantly monitored with simple and widely available ecological indicators. Such indicators should be easy to obtain and use, predictably sensitive and responsive to environmental impacts, and easy to interpret [1,2,3,4]. These indicators include parameters of animal condition, e.g., body size and weight, which should be constantly monitored as part of basic hunting management. Body weight, or carcass weight in the case of large game, is an easily accessible indicator of the condition of the population. Body weight also provides important information on the dynamics of changes in individual quality that determines the scope of procedures employed in the hunting management of game populations. In wild animals, body weight and size are directly associated with the physiological processes of the organism that significantly influence the individual condition of animals. In turn, the individual condition of game animals reflects the appropriate density in a hunting ground and determines further trends in population management [5,6]. As a rule, large and heavy males have preferential access to females and are more often chosen by females for mating. Larger and heavier females are characterized by higher reproductive success, longer lifespan, and earlier breeding, making them more likely to rear offspring [7,8,9]. This is also observed in other young animals, whose large size and body weight ensure a higher chance of surviving the first winter [10,11]. Larger specimens are also characterized by lower predation-related mortality and lower susceptibility to disease [12].

Animal body weight is mainly determined by body size and accumulated fat stores [6,13]. Parameters of body weight and condition are strongly dependent on environmental factors, with climatic conditions, food resources, population density, animal age, and seasonality being the primary determinants [14]. Body weight undergoes seasonal variations and depends on many factors, e.g., physiological states, such as pregnancy or parasite infestation [15,16,17,18]. The body weight of hares is also influenced by transformations of ecosystems, such as intensification of agriculture with the establishment of large-scale plantations. This type of simplified farming impairs the heterogeneity of agroecosystems, which provide the basic habitat for hares [19,20,21,22]. Given the relationship between environmental factors, body weight, and individual condition, the variability in these indicators can be used as a predictor of individual quality and as an accessible marker of the adaptation of a population to trophic habitat conditions.

Hares are closely related to field ecosystems. Although more than 50 species are known worldwide, only three representatives of the hare family exist in Europe: brown hare (*Lepus europaeus*), white-tailed hare (*Lepus timidus*), and wild rabbit (*Oryctolagus cuniculus*). Among these species, the brown hare is the most important, with a distribution range extending from the Pyrenees to Lake Baikal [23]. In ecological research on hare population management, the age of harvested animals must often be assessed [24,25]. The most commonly used method to determine the age of hares is palpation to detect the presence or absence of the Stroh sign (cartilaginous thickening of the ulnar epiphysis). Such an assessment facilitates discrimination between young (up to 1 year old) and adult (more than 1 year old) hares [23,26]. As suggested by Kauhal and Soveri [27], who compared three methods of hare age assessment (palpation, radiographic method, and lens weight), palpation is more useful than the radiographic method. The authors found that determination of eye lens weight and palpation are the most suitable methods of age assessment, yielding similar results.

## 2. Aim and Research Hypothesis

Owing to objective problems associated with field research, quick and precise assessment of the age of hares is difficult in practice. Hence, we developed an algorithm for classification of hares into two age classes: juveniles (up to 1 year old) and adults (more than 1 year old) based on body weight, which can be determined by both examination of live animals and postmortem analysis. Hare age assessment based on lens weight is the most precise method; however, it is difficult to implement on hunting grounds and can only be performed after culling. Therefore, we decided against employing this approach, assuming a strong correlation between eye-lens weight and hare-carcass weight, as demonstrated by Meomartino et al. [28]. Considering this correlation, we assumed that hares can be classified into one of the two age groups based on carcass weight alone and using an appropriate logistic model.

## 3. Materials and Methods

### 3.1. Animals and Research Area

**Figure 1:**
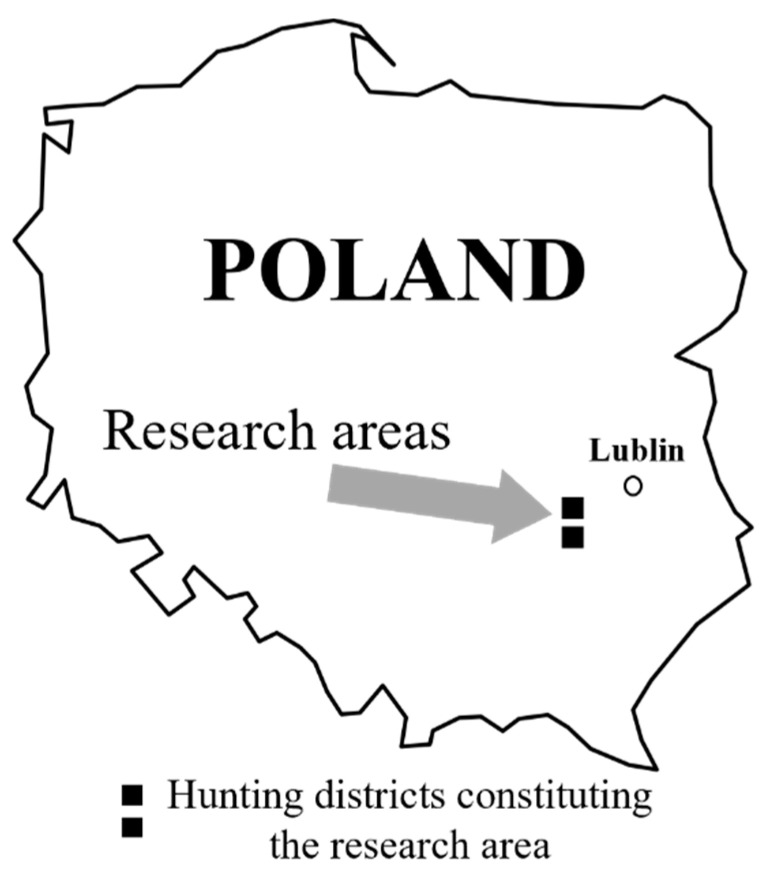
Location of the research area.

The research involved 297 hares culled in two hunting districts located in Lublin Upland, where the species is still hunted (Figure 1). Both districts are characterized by a high density of brown hares, thus making hunting possible without a detrimental effect on the population. The harvesting rate in these districts during the last two hunting seasons was approximately 5 hares per 100 ha area. The research was conducted during group hunting in November and December 2019, in accordance with provisions of Polish hunting laws. Due to the COVID-19 restrictions imposed in 2020, the animals were culled by individual hunters in compliance with the decision of the Minister of Climate and Environment of 15 December 2020 (DLŁ-ZŁ.4142.1.2020).

### 3.2. Methods and Measurements

The body weight of the animals was measured immediately after culling. The hares were weighed with an accuracy of 0.1 kg using a Kern HCB20K10 portable balance (Kern & Sohn, Albstadt, Germany). Age was determined by assessing the presence or absence of the Stroh sign; this allowed for discrimination between juvenile (up to 1 year old) and adult (more than 1 year old) animals. The sex of the animals was determined based on the appearance of secondary sexual characteristics [23,26].

Additionally, the left eyeball was dissected from each individual and preserved in 10% buffered formalin. The lens was removed from each eyeball in laboratory conditions to determine the age of the hare based on the weight of the dried lens and to verify the results of the field method. The lenses were dried to constant weight in an SML 32 laboratory dryer (Zelmer, Poznań, Poland) at 100 °C for 24 h [29]. After drying, the lenses were weighed on an analytical balance (Pioneer OHAUS, Nänikon, Switzerland) with an accuracy of 0.001 g. This method is based on the observation that the eye-lens weight increases relatively intensively during the first year of life, whereas a lower increase is recorded in the following years. In accordance with this principle and based on the data obtained in the study, the animals were categorized as juveniles or adults, with an eye-lens-weight cutoff value of 290 mg [29,30,31,32,33].

## 4. Analysis

The hares were divided into two age groups according to the carcass weight of individual animals. Due to the lack of sexual dimorphism in terms of body weight of females and males, the animals were not grouped according to sex. Body weight and dry lens weight were analyzed in the separate age groups in terms of the following characteristics: mean value, standard deviation, minimum, and maximum. We assessed the strength of the relationships between the examined variables, using the Pearson correlation coefficient; the results are illustrated in a scatterplot with a fitted regression line.

Using logistic regression, a model was constructed to assess the age of hares based on body weight. For comparison with the current age-assessment method, a logistic regression was also performed to classify the hares based on dry lens weight.

## 5. Results

Body weight and dry lens weight were analyzed separately in the two age groups. Body weight ranged from 3.3 to 4.4 kg in the juvenile group and from 4.0 to 5.5 kg in the adult group. The mean value of this parameter was 3.95 kg in the juvenile group and 4.56 kg in the adult group, with similarly low standard deviations of 0.23 and 0.26 kg, respectively (Table 1).

The dry lens weight ranged from 0.17 to 0.40 g in the juvenile group and from 0.22 g to 0.56 g in the adult group. The mean dry lens weight in the juvenile group was 0.25 g, with a standard deviation of 0.03 g. The mean dry lens weight in the adult group was 0.38 g, with a standard deviation of 0.06 g (Table 1).

**Figure 2:**
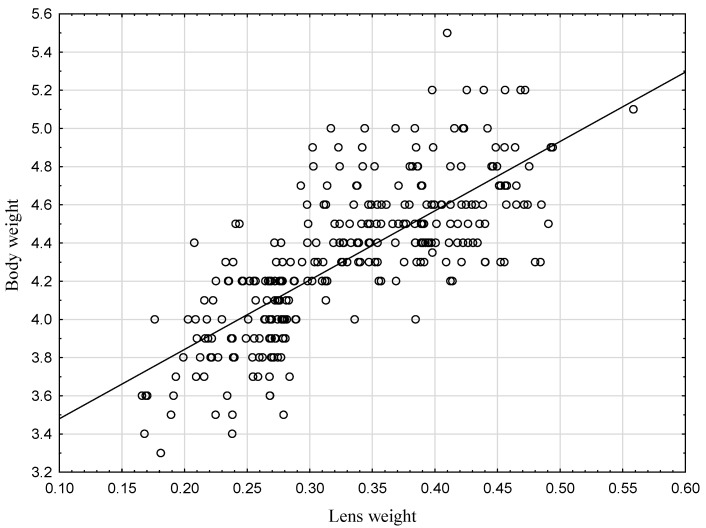
Scatterplot of body weight and dry lens weight with a fitted regression line.

Next, the Pearson correlation coefficient was calculated, and a very strong positive correlation was found between body weight and dry lens weight (Pearson correlation coefficient *r* = 0.75, *p* < 0.001) (Figure 2). Such a strong correlation suggests that the age of hares can be determined based not only on dry lens weight but also body weight. A model was generated by using logistic regression to estimate the age of hares based on body weight.

(1) $$P\left(Y=A|WEIGHT\right)=\frac{e^{-70.1836+16.6019\cdot WEIGHT}}{1+e^{-70.1836+16.6019\cdot WEIGHT}}$$

**Figure 3:**
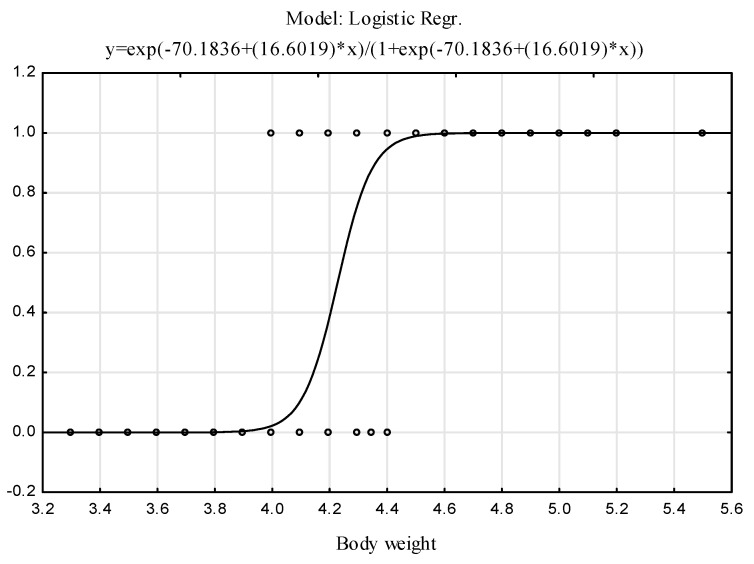
Fitted logit function and observed values (1 = A, 0 = J).

This relationship expresses the probability of classification of an individual into group A (adult hares) based on body weight. If the probability is at least 0.5, the model classifies such an individual as an adult animal (A); otherwise, the animal is assigned to the juvenile group (J). The logit function plot is presented in Figure 3, and the model parameters are shown in Table 2.

This model correctly classified 92.59% of the hares into the two age groups according to their body weight, with 93.39% and 92.05% accuracy for juveniles (J) and adults (A), respectively (Table 3). The odds ratio of correct classifications to the adult group based on body weight was 163 times larger than that of incorrect classifications.

**Figure 4:**
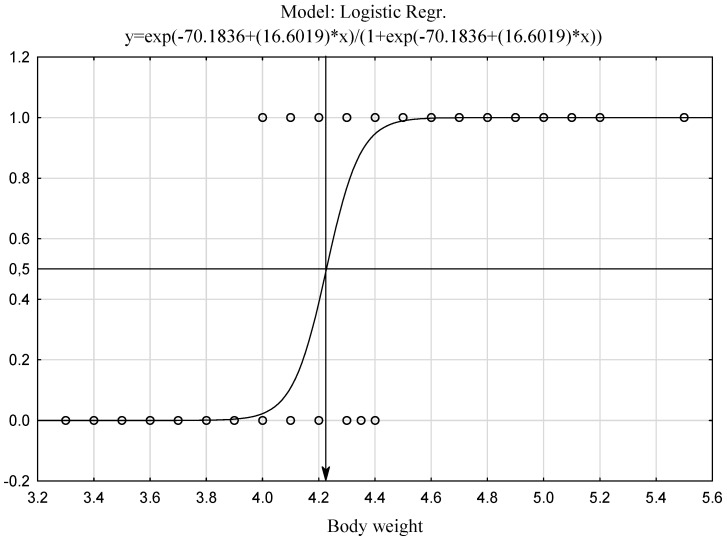
Cutoff point for classification of cases based on body weight (1 = A, 0 = J).

The presented model classifies hares into the adult group when their body weight exceeds 4.227 kg (Figure 4). To compare the proposed body-weight-based model of age classification with the current method based on the dry lens weight, a logistic model was generated.

(2) $$P\left(Y=A|LENS\right)=\frac{e^{-20.2314+68.2144\cdot LENS}}{1+e^{-20.2314+68.2144\cdot LENS}}$$

**Figure 5:**
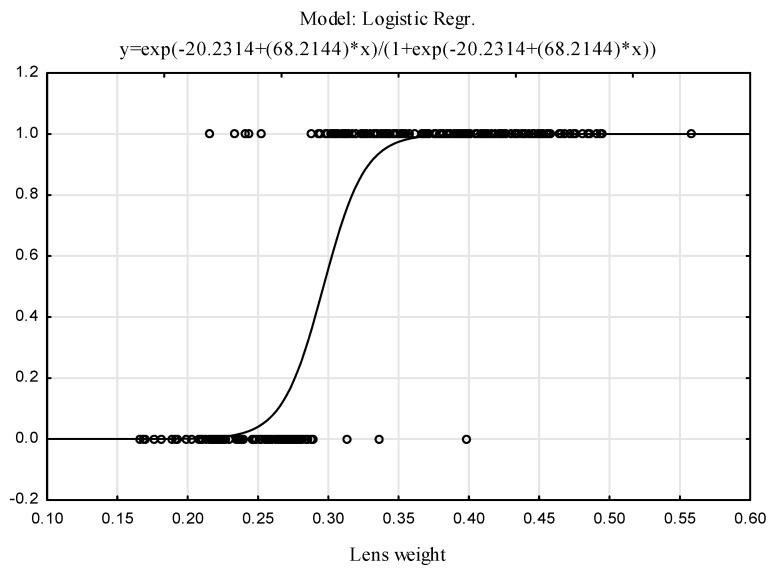
Fitted logit function and observed values (1 = A, 0 = J).

The logit function plot is presented in Figure 5, and the model parameters are shown in Table 4.

This model correctly classified 96.30% of the hares into two age groups according to their lens weight, including 97.52% of the juvenile hares (J) and 95.45% of the adult hares (A) (Table 5).

**Figure 6:**
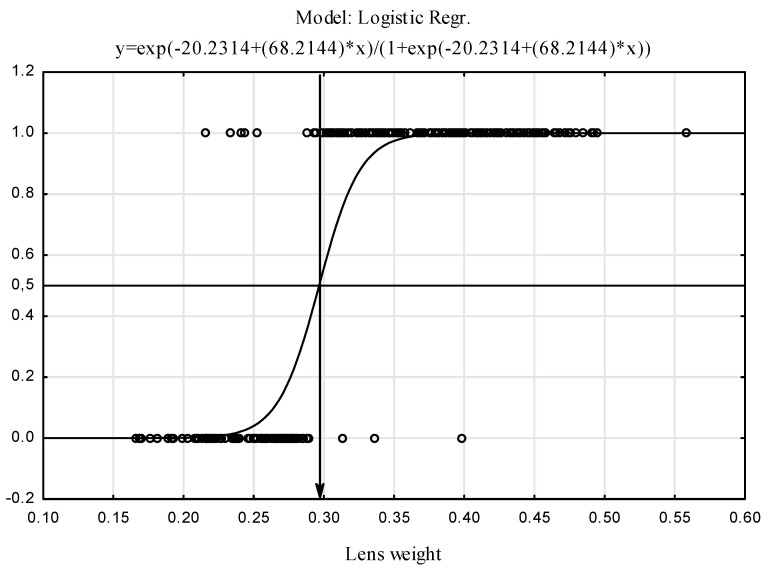
Cutoff point for classification of cases based on dry lens weight (1 = A, 0 = J).

The generated model classifies hares into the adult group when their dry lens weight exceeds 0.297 kg (Figure 6). These results are consistent with those obtained by the lens-weight-based age assessment model reported by Pintur [34]. The two models achieved a similar percentage of correct classifications, which indicates the potential of our alternative hare age-assessment model based on body weight.

## 6. Discussion

To identify population trends in wild animals, many parameters must be determined, with the age of individual animals being one of the most important factors. Knowing the age of individual animals allows for population prediction in ecosystems exposed to environmental factors. Determining the age of hares is a complicated task. The Stroh method has been used for many years both in population management and scientific research. It consists of palpation-based examination of cartilage thickening on the outer side of the ulna at a distance of approximately 1 cm from the wrist joint. Since the thickening disappears at the age of 8–9 months, this method allows for discrimination between juvenile and adult (more than 1 year old) animals [26,35]. However, the results of this method do not facilitate precise determination of age. An advantage of this approach is the possibility of examining live animals; a drawback is that it requires considerable experience in determining the presence or absence of the Stroh sign.

In scientific research, the age of animals is often assessed based on dry lens weight. Laboratory weighing of lenses can help to discriminate between juvenile (up to 1 year old) and adult (more than 1 year old) individuals [22,29,30,31,32,33]. Since the lens weight gain in the first year of life is more intense, the weight data indicate the time of birth [36]. In older individuals, lens weight gain is much slower, although it is possible to estimate the age of animals and distinguish the subsequent years of life in individuals more than 1 year old [29,31,34]. Despite its accuracy, the main drawback of this method is that assessment can only be performed on culled hares (postmortem). Therefore, we constructed a body-weight-based age-assessment method as an alternative approach. The results of the present research facilitated generation of a logistic model, which showed 93.39% and 92.05% accuracy in the age assessment in juvenile and adult hares, respectively. The key advantages of the proposed method are its simplicity; the possibility of assessing live animals with minimized invasiveness, even in comparison with the Stroh method; and its high efficiency.

## 7. Conclusions

The results of the present study facilitated the development of an age-classification method for hares based on body weight. The proposed approach is innovative, as it allows for determination of the age of not only culled (postmortem) but also live hares. The method is easy and does not require laboratory tests; hence, the results can be used immediately following evaluation. This method allows for hares to be assigned to one of two age groups (juveniles and adults). The developed logistic regression model produced a body-weight cutoff value of 4.227 kg. The estimated accuracy of the model is 97.52% and 95.45% for juvenile and adult hares, respectively. Such high accuracy indicates that the proposed approach can be widely used both in population management and scientific research. However, even in the European region, depending on latitude, hare size and, thus, body weight, vary. Therefore, in the case of hares from the southern and northern ends of the continent, the model should be verified. Nevertheless, the proposed method can achieve similar accuracy when adjusted for varying body-weight distributions.

## Figures and Tables

**Table 1:** Body weight and dry lens weight in the two age groups.

Item	Age		Mean	*p*-Value
	Juveniles (*n* = 121)	Adults (*n* = 176)		
Body weight, kg	3.95 ± 0.23	4.56 ± 0.26	4.31 ± 0.39	0.023
Dry lens weight, kg	0.25 ± 0.03	0.38 ± 0.06	0.33 ± 0.08	0.012

**Table 2:** Parameter evaluation in the logistic regression model in relation to body weight.

*N* = 297	Total Loss: 55.7128 Chi^2^(1) = 290.06 *p* = 0.0000; Modeled *p*, Age = A	
	Constant B0	Body Weight
Estimation	−70.1836	16.6019
Standard error	11.3284	2.6707
t (295)	−6.1953	6.2164
*p*	0.000000002	0.000000002

**Table 3:** Classification of cases in the logistic model in relation to body weight.

Observed	Classification of Cases; Odds: 163.45; % Correctness: 92.59%		
	Predicted (J)	Predicted (A)	Percent Correctness
Juveniles	113	8	93.39
Adults	14	162	92.05

**Table 4:** Parameter evaluation in the logistic regression model in relation to dry lens weight.

*N* = 297	Total Loss: 59.7183 Chi^2^(1) = 282.05 *p* = 0.0000, Modeled *p*, Age = A	
	Constant B0	Lens Weight
Estimation	−20.2314	68.2144
Standard error	2.7437	9.4179
t (295)	−7.3738	7.2431
*p*	0.000000000002	0.000000000004

**Table 5:** Classification of cases in the logistic model in relation to dry lens weight.

Observed	Classification of Cases; Odds: 826.00; % Correct: 96.30%		
	Predicted	Predicted	Percent Correctness
	J	A	
Juveniles	118	3	97.52
Adults	8	168	95.45

## Data Availability

The data presented in this study are available upon request from the corresponding author.
